# Supplementary material for: Carbon dioxide capture and efficient fixation in a dynamic porous coordination polymer
Source: Nat Commun. 2019 Sep 25;10:4362. doi: 10.1038/s41467-019-12414-z (PMC6761133; doi:10.1038/s41467-019-12414-z)
Supplement: Supplementary file 1 — Supplementary Information [file 41467_2019_12414_MOESM1_ESM.pdf]

Supplementary Information

Carbon Dioxide Capture and Efficient Fixation in a Dynamic Porous Coordination Polymer

Wu et al.

Supplementary Methods

Synthesis of H_3tca ligand.

(a) 4,4',4''-triacetyl Triphenylamine: To the solution of triphenylamine (1.0 g, 4.1 mmol) and 1.8 g (13.5 mmol) $AlCl_3$ in 150 mL dry CH_2Cl_2, 2.0 mL (28.28 mmol) acetyl chloride in 50 mL dry CH_2Cl_2 was added dropwisely. Then the reaction was carried out at room temperature for 12 hours and 100 mL water was added. The aqueous layer was further extracted with CH_2Cl_2 (3×150 mL); the combined organic layers were dried by Na_2SO_4. The solution was concentrated under reduced pressure to give the crude product, which was purified by flash column chromatography on silica gel (1: 10 hexanes–CH_2Cl_2) to afford a yellow solid (0.94 g, 62.3%). ^{1}H NMR ($CDCl_3$, *ppm*): δ 7.90 (d, 6H, J = 8.0 Hz), 7.15 (d, 6H, J = 8.0 Hz), 2.58 (s, 9H).

(b) 4,4',4''-tricarboxyl Triphenylamine (H_3tca): 3 mL Br_2 were added dropwisely to the solution of NaOH (7 g, 0.18 mol) in 30 mL water on ice bath, and further stirred for 20 min. The solution were dumpage to a isobarically funnel and were added dropwisely to a solution of 4,4',4''-triacetyl Triphenylamine (2.0 g, 5.4 mmol) in 50 mL 1, 4-Dioxane on 45 ℃ during 5 h. Then the mixture was put on ice-bath, saturated hydroxylamine HCl was added to deoxidize excessive sub-bromo-sodium. The solution was acidified by muriatic acid and the solid product was filtered and dried under vacuum. The crude was recrystallized from acetic acid to afford pure products as a white solid. (1.79 g, 88.2%). ^{1}H NMR (DMSO-d_6, *ppm*): δ 12.75 (s, 3H), 7.91 (d, 6H, J = 8.8 Hz), 7.14 (d, 6H, J = 8.8 Hz).

Synthesis of (E)-1,2-di(pyridin-4-yl)diazene (dpa) ligand.

A solution of 4-aminopyridine (5.0 g) was dissolved in 100 mL of water. The solution was cooled and then added drop wise to 300 mL of a 10% NaOCl solution at 0 °C. The mixture was stirred at 0 °C for 30 minutes. The orange precipitate was filtered. The crude solid was dissolved in CH_2Cl_2. Black solid was removed *via* filtration was collected and the solvent was removed *via* rotary evaporation. The orange solid was then recrystallized from water. (3.17 g, 64.8%). ^{1}H NMR (DMSO-d_6, *ppm*): δ 7.82 (dd, 4H, J = 4.0, 8.0 Hz), 8.90 (dd, 4H, J = 4.0, 8.0 Hz).

Single-crystal X-ray Diffraction Analyses. Intensities were collected on a Rigaku XtaLAB AFC10 diffractometer using a VariMax Mo Optic with Mo-Kα (λ = 0.71073 Å). The structure was solved by direct methods and refined on F^2 by full-matrix least-squares methods with SHELXTL *version* 2018/3. Non-hydrogen atoms of the ligand backbones were refined anisotropically. Hydrogen atoms within the ligand backbones were fixed geometrically at calculated positions and allowed to ride on the parent non-hydrogen atoms.

Zn-DPA·2H_2O. As-synthesized single crystals were put into paraton oil. One crystal suitable for diffraction measurement was quickly transferred on the diffractometer using MiTeGen MicroMount at183 K.

Refinement details of Zn-DPA·2H_2O. All framework atoms were located and refined anisotropically. The several areas of residual electron density were found inside the framework pores and were assigned as disordered water molecules (O1WA, O2WA, O3WA, O1WB, O2WB, O1WC, O2WC). PART instruction (PART 1 for O1WA, O2WA, O3WA; PART 2 for O1WB, O2WB; PART 3 for O1WC, O2WC), and one thermal restraint (ISOR) on water molecule were also used for the structural refinement. Their chemical occupancy was first refined and then fixed to a value of 0.5 for O1WA and 0.25 for the other O sites, that equals the total sum number of two water molecules per $Zn_{1.5}$, as consistent with the TGA and elemental analysis results.

*Response to A and B level check cif alerts for **Zn-DPA·2H_2O** single crystal:*

Zn-DPA·2H_2O.cif contains one alert B as follows.

Alert level B

PLAT601_ALERT_2_B Structure Contains Solvent Accessible VOIDS of . 137 Ang**3

Response: The structure still contains a large solvent channel except the occupation of two water molecules. The remaining voids might be suitable to take up further solvent, but none was seen in difference Fourier syntheses and squeezing with program PLATON did not give significant improvement or change.

Selective bond distance (Å) and angle (°) in Zn-DPA·2H_2O.

Selective bond distance (Å): Zn(1)–O(6) 2.0901(17), Zn(1)–O(3A) 2.0899(18), Zn(1)–O(1B) 2.0904(17), Zn(2)–O(2B) 1.9706(19), Zn(2)–O(3A) 2.1543(18), Zn(2)–O(4A) 2.141(2), Zn(2)–O(5) 1.957(2), Zn(2)–N(2) 2.048(2); Selective bond angle (°): O(5E)–Zn(1)–O(3A) 180.00(9), O(3E)–Zn(1)–O(6) 86.45(7), O(3A)–Zn(1)–O(6) 93.55(8), O(3E)–Zn(1)–O(6D) 93.55(7), O(3A)–Zn(1)–O(6D) 86.45(7), O(6D)–Zn(1)–O(6) 180.0, O(3E)–Zn(1)–O(1B) 91.77(7), O(3A)–Zn(1)–O(1B) 88.23(7), O(6)–Zn(1)–O(1B) 94.48(7), O(6D)–Zn(1)–O(1B) 85.52(7), O(3E)–Zn(1)–O(1C) 88.23(7), O(3A)–Zn(1)–O(1C) 91.77(7), O(6)–Zn(1)–O(1C) 85.52(7), O(6D)–Zn(1)–O(1C) 94.48(7), O(1B)–Zn(1)–O(1C) 180.00(8), O(5)–Zn(2)–O(2B) 108.53(9), O(5)–Zn(2)–N(2) 93.65(9), O(2B)–Zn(2)–N(2) 100.16(8), O(5)–Zn(2)–O(4A) 104.53(10), O(2B)–Zn(2)–O(4A) 141.78(9), N(2)–Zn(2)–O(4A) 96.30(8), O(5)–Zn(2)–O(3A) 109.49(7), O(2B)–Zn(2)–O(3A) 90.10(8), N(2)–Zn(2)–O(3A) 150.31(8), O(4A)–Zn(2)–O(3A) 60.70(7). Symmetry code A: *x*, -1+*y*, *z*; B: -0.5+*x*, -0.5+*y*, *z*; C: 1-*x*, 1-*y*, 1-*z*; D: 0.5-*x*, 0.5-*y*, 1-*z*; E: 0.5-*x*, 1.5-*y*, 1-*z*.

Zn-DPA. As-synthesized single crystals were air-dried. One single crystal suitable for the diffraction measurement was put into a glass capillary. The capillary was connected to handmade gas pressure handling unit, and was evacuated (below 10^{-2} Pa) at 120 °C overnight. Then, the capillary was sealed using small torch flame with keeping vacuum condition inside the capillary. The sealed capillary was mount on the diffractometer at 183 K.

Refinement details of Zn-DPA. All framework atoms were located and refined anisotropically.

Response to A level check cif alerts for ***Zn-DPA*** *single crystal:*

Zn-DPA.cif contains one alert A as follows.

Alert level A

PLAT602_ALERT_2_A VERY LARGE Solvent Accessible VOID(S) in Structure ! Info

Response: Application of procedure SQUEEZE (program PLATON) did not bring about a significant improve of refinement results and therefore was not retained for the final refinement. This might be due to the existence of a large solvent channel in the structure itself.

Selective bond distance (Å) and angle (°) in Zn-DPA.

Selective bond distance (Å): Zn(1)–O(1) 2.1051(15), Zn(1)–O(4B) 2.0867(14), Zn(1)–O(6A) 2.0784(15), Zn(2)–O(6A) 2.1625(15), Zn(2)–O(5A) 2.1230(17), Zn(2)–O(3B) 1.9570(16), Zn(2)–O(2) 1.9404(16), Zn(2)–N(2) 2.0627(17); Selective bond angle (°): O(6D)–Zn(1)–O(6A) 180.00(11), O(6D)–Zn(1)–O(4E) 87.91(6), O(6A)–Zn(1)–O(4E) 92.09(6), O(6D)–Zn(1)–O(4B) 92.09(6), O(6A)–Zn(1)–O(4B) 87.91(6), O(4E)–Zn(1)–O(4B) 180.00(5), O(6D)–Zn(1)–O(1C) 93.32(6), O(6A)–Zn(1)–O(1C) 86.68(6), O(4E)–Zn(1)–O(1C) 94.10(6), O(4B)–Zn(1)–O(1C) 85.90(6), O(6D)–Zn(1)–O(1) 86.68(6), O(6A)–Zn(1)–O(1) 93.32(6), O(4E)–Zn(1)–O(1) 85.90(6), O(4B)–Zn(1)–O(1) 94.10(6), O(1C)–Zn(1)–O(1) 180.0, O(2)–Zn(2)–O(3B) 110.00(8), O(2)–Zn(2)–N(2) 94.24(7), O(3B)–Zn(2)–N(2) 97.81(7), O(2)–Zn(2)–O(5A) 108.83(8), O(3B)–Zn(2)–O(5A) 137.74(8), N(2)–Zn(2)–O(5A) 95.32(7), O(2)–Zn(2)–O(6A) 111.58(6), O(3B)–Zn(2)–O(6A) 89.70(6), O(2)–Zn(2)–O(6A) 148.83(7), O(5A)–Zn(2)–O(6A) 60.67(6). Symmetry code A: *x*, 1+*y*, *z*; B: -0.5+*x*, 0.5+*y*, *z*; C: 0.5-*x*, 0.5-*y*,-*z*; D: 0.5-*x*, -0.5-*y*, -*z*; E: 1-*x*,-*y*,-*z*.

Zn-DPA·2CO_2. As-synthesized single crystals were air-dried. One single crystal suitable for the diffraction measurement was put into a glass capillary. The capillary was connected to handmade gas pressure handling unit, and was evacuated (below 10^{-2} Pa) at 120 °C overnight. Then, CO_2 was slowly introduced into the capillary until the pressure reaching 101 *k*Pa at 195 K. After 10 min at 195 K under 101 *k*Pa CO_2 atmosphere, the glass capillary was sealed using small torch flame. The sealed capillary was mount on the diffractometer at 183 K.

Refinement details of Zn-DPA·2CO_2. All framework atoms were located and refined anisotropically. Three isolated areas (here we name them as A, B, and C, which corresponding to site I, III, and II in the manuscript)

of residual electron density were observed inside the framework pores and were assigned as accommodated CO_2 molecules. Because CO_2 molecule at C (site II) was disordered into two positions (here we name them as C and CC), PART instruction was used (PART 1 for "O1C-C1C-O2C"; PART 2 for "O1CC-C1CC-O2CC"). Some thermal and structural restraints (ISOR, SADI, DFIX, DANG) were also used for the structural refinements of CO_2 molecules at B and C (site II and III). As the sorption measurements showed that this compound adsorbed $2CO_2$ molecules per $Zn_{1.5}$ under 101 *k*Pa CO_2 atmosphere at the measurement temperature, we can expect the sum total number of CO_2 at A, B, C, and CC (site I, III, II) to be two. Therefore, Free variables were introduced for the occupancy refinements of all CO_2 molecules with using SUMP instruction (SUMP 2 0.00001).

Selective bond distance (Å) and angle (°) in Zn-DPA·2CO$_2$.

Selective bond distance (Å): Zn(1)–O(1) 2.1022(14), Zn(1)–O(3A) 2.0940(14), Zn(1)–O(5B) 2.0870(14), Zn(2)–O(6B) 1.9711(15), Zn(2)–O(4A) 2.1092(16), Zn(2)–O(3A) 2.1902(15), Zn(2)–O(2) 1.9434(15), Zn(2)–N(2) 2.0536(17); Selective bond angle (°): O(5C)–Zn(1)–O(5B) 180.00(5), O(5C)–Zn(1)–O(3D) 88.49(6), O(5B)–Zn(1)–O(3D) 91.51(6), O(5C)–Zn(1)–O(3A) 91.51(6), O(5B)–Zn(1)–O(3A) 88.49(6), O(3D)–Zn(1)–O(3A) 180.0, O(1E)–Zn(1)–O(5C) 94.44(6), O(5B)–Zn(1)–O(1E) 85.56(6), O(3D)–Zn(1)–O(1E) 93.51(6), O(3A)–Zn(1)–O(1E) 86.49(6), O(5C)–Zn(1)–O(1) 85.56(6), O(5B)–Zn(1)–O(1) 94.44(6), O(3D)–Zn(1)–O(1) 86.49(6), O(3A)–Zn(1)–O(1) 93.51(6), O(1)–Zn(1)–O(1E) 180.0, O(2)–Zn(2)–O(6B) 111.42(7), O(2)–Zn(2)–N(2) 94.64(7), O(6B)–Zn(2)–N(2) 97.61(7), O(2)–Zn(2)–O(4A) 105.45(7), O(4A)–Zn(2)–O(6B) 138.83(7), N(2)–Zn(2)–O(4A) 97.03(7), O(2)–Zn(2)–O(3A) 112.47(6), O(6B)–Zn(2)–O(3A) 88.12(6), N(2)–Zn(2)–O(3A) 148.10(6), O(4A)–Zn(2)–O(3A) 60.68(6). Symmetry code A: *x*, -1+*y*, *z*; B: 0.5+*x*, -0.5+*y*, *z*; C: 1-*x*,1-*y*,1-*z*; D:1.5-*x*,1.5-*y*,1-*z*; E: 1.5-*x*, 0.5-*y*, 1-*z*.

Computational Details.

The experimentally observed adsorption isotherm of CO_2 suggests that 16 molecules of CO_2 can be absorbed into one unit cell of Zn-**DPA** at the saturated limit. To locate the adsorption positions of these CO_2 molecules,

we carried out canonical Monte-Carlo (MC) simulations,[1] as implemented in RASPA,[2] using Lennard-Jones (LJ) potentials to describe the Van der Waals interaction of CO_2 with Zn-**DPA** framework. The LJ parameters were taken from the standard universal force field (UFF)[3] and TraPPE[4] for Zn-**DPA** and CO_2, respectively, where the Lorentz-Berthelot mixing rules were used for different atoms. The electrostatic interaction was evaluated with the Ewald summation method, where the DDEC atomic charges[5,6] were used. The crystal structure of Zn-**DPA** containing 8 CO_2 molecules in one unit cell has been observed in experiment. Using that structure, we started the MC simulation to find adsorption positions of other CO_2 molecules by placing 64 CO_2 molecules, which correspond to adsorption of 16 CO_2 molecules into one unit cell, in a simulation box of 2×2×2 supercell of Zn-**DPA**. In the MC simulation, the first 2×10^5 cycles were consumed for obtaining equilibration and then 5×10^5 cycles were used for obtaining distribution of CO_2 molecules at room temperature. In the final configuration, we found that the positions of CO_2 molecules could be classified into three groups, suggesting that there are three possible sites, namely sites I, II, and III, for CO_2 adsorption to Zn-**DPA**, where the site I is the same as the CO_2 positions observed in experiment (Supplementary Fig. 13). The Fo-Fc contoured Fourier Map of the crystal structure also implied some residual electron densities at the positions similar to the computational CO_2 adsorption sites II and III. The model CO_2 molecules were installed on the sites II and III, and their positions and occupancies were further refined in the SCXRD analysis and the obtained results are consistent with the computational results about CO_2 positions at the sites II and III. Considering the symmetry of Zn-**DPA**, each of these three sites has 8 equivalent positions. The final configuration of the above MC simulation was used as the initial structure for performing geometry optimization with density functional theory (DFT).

The adsorption energy was calculated using DFT method with periodic boundary conditions as implemented in the Vienna Ab initio Simulation Package (VASP 5.4.1).[7,8] The Perdew-Burke-Ernzerhof functional[9] with Grimme's semi-empirical "D3" dispersion term[10] (PBE-D3) was employed in these calculations. The plane wave basis sets with an energy cutoff of 500 eV were used to describe valence electrons and the projector-augmented-wave pseudopotentials[11,12] were used to describe core electrons. The criterion of atomic force for geometry optimization was set to be 0.002 eV/Å. The Brillouin zone was sampled by a Γ-point in these calculations.

The binding energy (*BE*) of CO_2 with Zn-**DPA** was calculated with equation 1;

$$BE = [E(\mathbf{Zn\text{-}DPA}\cdot n\mathrm{CO_2})_{eq} - E(\mathbf{Zn\text{-}DPA})_{eq} - nE(\mathrm{CO_2})_{eq}]/n \quad (1)$$

where $E(\mathbf{Zn\text{-}DPA}\cdot n\mathrm{CO_2})_{eq}$ is the total energy of **Zn-DPA** with *n* molecules of CO_2 per one unit cell in equilibrium structure (eq), $E(\mathbf{Zn\text{-}DPA})_{eq}$ and $E(\mathrm{CO_2})_{eq}$ are the total energies of empty Zn-**DPA** and one free CO_2 molecule, respectively, in their equilibrium structure. Similar calculations were carried out to obtain binding energies of N_2 and substrate epoxide with Zn-**DPA**. The *BE* was decomposed into the deformation energy [E_{DEF}(H)] of Zn-**DPA**, the interaction energy [E_{INT}(H-G)] between Zn-**DPA** (H) and CO_2 molecule (G) and the interaction energy [E_{INT}(G-G)] among adsorbed CO_2 molecules. These energy terms were calculated with equations 2~4;

$$E_{DEF}(\mathrm{H}) = [E(\mathbf{Zn\text{-}DPA})_{dis} - E(\mathbf{Zn\text{-}DPA})_{eq}]/n \quad (2)$$

$$E_{INT}(\mathrm{H\text{-}G}) = [E(\mathbf{Zn\text{-}DPA}\cdot n\mathrm{CO_2})_{eq} - E(\mathbf{Zn\text{-}DPA})_{dis} - E(n\mathrm{CO_2})_{dis}]/n \quad (3)$$

$$E_{INT}(\mathrm{G\text{-}G}) = [E(n\mathrm{CO_2})_{dis}]/n - E(\mathrm{CO_2})_{eq} \quad (4)$$

where $E(\mathbf{Zn\text{-}DPA})_{dis}$ and $E(n\mathrm{CO_2})_{dis}$ are the total energies of empty **Zn-DPA** and nCO_2 cluster taking the same distorted structures (dis) as those in Zn-**DPA**·nCO_2.

To make analysis of CO_2 interaction with Zn-**DPA**, three cluster models (CMs) were constructed for the sites I, II, and III, using the PBE-D3 optimized structure. These cluster models were further decomposed into several small cluster models (SCMs) to elucidate important interactions that stabilise CO_2 adsorption at these sites, as shown in Supplementary Fig. 14. The dangling bonds in these CMs and SCMs were capped with hydrogen atoms.

Supplementary Table 1. Crystallographic data and structural refinement summary for Zn-**DPA**·$2H_2O$, Zn-**DPA** and Zn-**DPA**·$2CO_2$.

	Zn-**DPA**·$2H_2O$	Zn-**DPA**	Zn-**DPA**·$2CO_2$
Empirical formula	$C_{26}H_{16}N_3O_8Zn_{1.5}$	$C_{26}H_{16}N_3O_6Zn_{1.5}$	$C_{28}H_{16}N_3O_{10}Zn_{1.5}$
Formula wt	596.47	564.47	652.49
Crystal System	Monoclinic	Monoclinic	Monoclinic
Space Group	*C2/c*	*C2/c*	*C2/c*
a (Å)	24.3603(11)	24.0983(13)	24.5697(14)
b (Å)	13.3966(3)	13.5396(4)	13.3294(3)
c (Å)	22.8922(11)	22.9142(12)	22.9612(13)
α (°)	90.00	90.00	90.00
β (°)	127.056(7)	127.451(8)	127.632(9)
γ (°)	90.00	90.00	90.00
V (Å^3)	5962.0(6)	5935.4(7)	5955.3(8)
Z	8	8	8
R_{int}	0.0582	0.0478	0.0534
μ(cm^{-1})	1.264	1.261	1.277
D_{calcd}/ Mg m^{-3}	1.329	1.263	1.456
F(000)	2416	2288	2640
Goodness of fit	1.102	1.041	1.048
Temperature (K)	183(2)	183(2)	183(2)
Reflections collected	8972	8998	8948
Independent reflections	7344	6561	7143
R (I > 2.00σ(I), all data)	0.0492	0.0474	0.0418
R_{w} (I > 2.00σ(I), all data)	0.1477	0.1015	0.1181

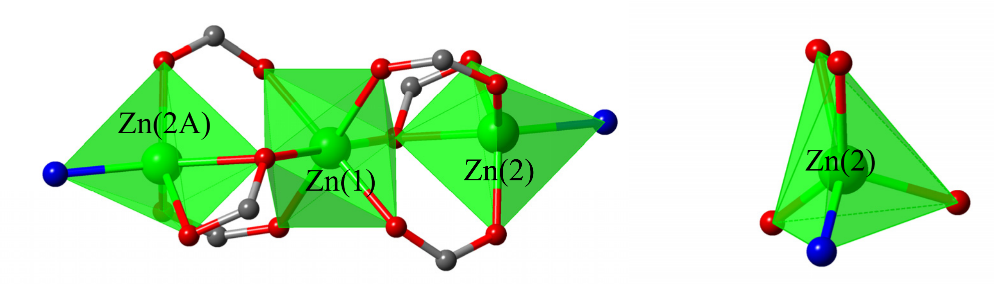

Supplementary Figure 1. Coordinated environment of the Zn^{2+} ions in Zn-**DPA**·$2H_2O$. The right picture represents the enlarged view of pseudo-tetragonal pyramid configuration of Zn(1) center. Symmetry codes: A 0.5-*x*, 0.5-*y*, 1-*z*.

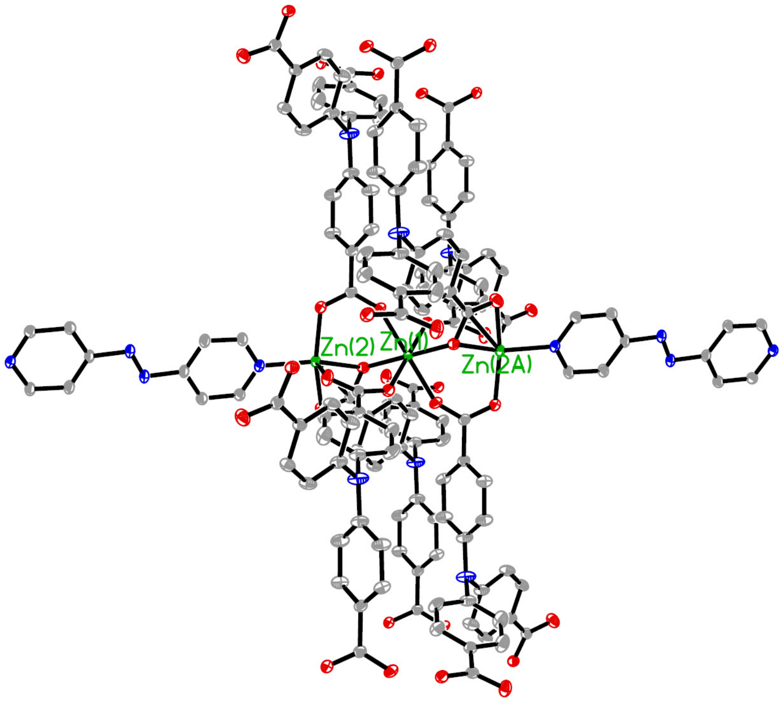

Supplementary Figure 2. The trinuclear $Zn_3(CO_2)_6$ cluster in Zn-**DPA**·$2H_2O$. Symmetry codes: A 0.5-*x*, 0.5-*y*, 1-*z*.

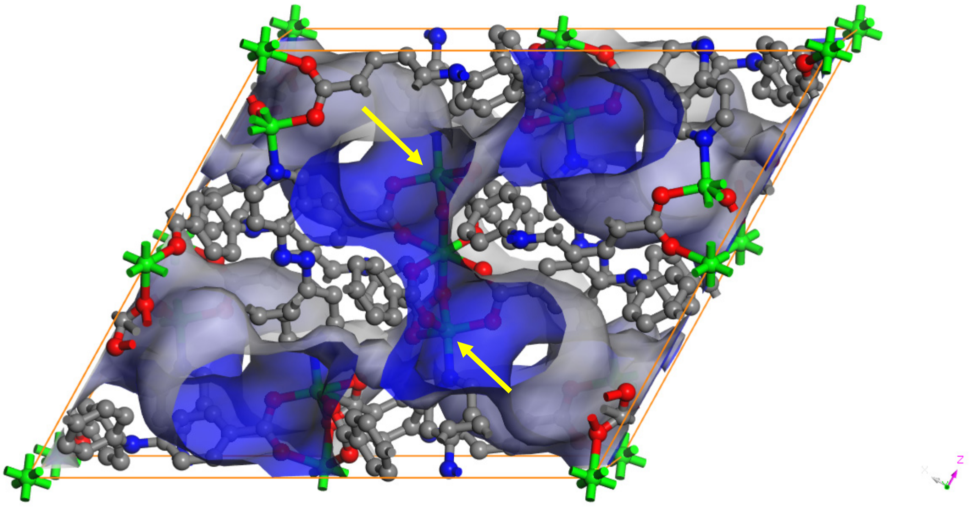

Supplementary Figure 3. Three-dimensionally connected channels in Zn-**DPA**·$2H_2O$ with the Connolly surfaces (Connolly radius: 1.6 Å) along *b*-axis. Water molecules are omitted for clarity. The yellow arrows indicate the free Lewis acid zinc centers.

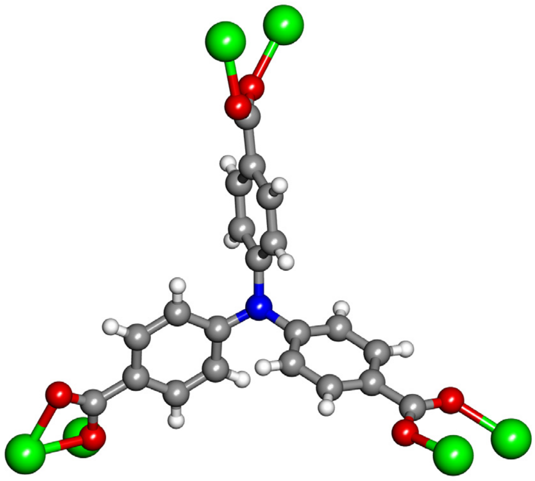

Supplementary Figure 4. Coordination modes of tca^{3-} anions in Zn-**DPA**·$2H_2O$.

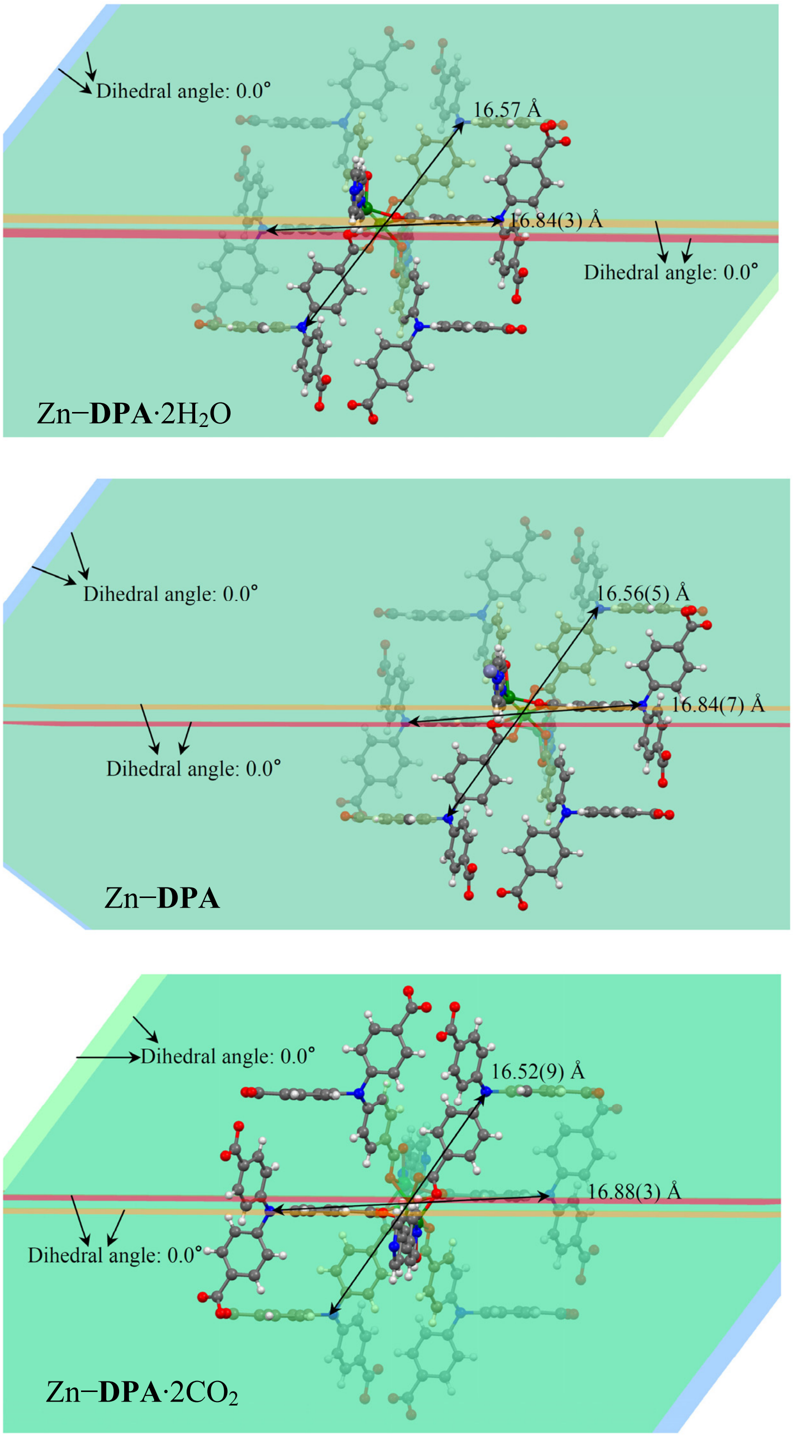

Supplementary Figure 5. Comparison of the dihedral angle between the phenyl rings of two tca^{3-} ligands located at the *trans* position of the $Zn_3(CO_2)_6$ cluster and the N···N distance between these ligands in Zn-**DPA**·$2H_2O$, Zn-**DPA** and Zn-**DPA**·$2CO_2$.

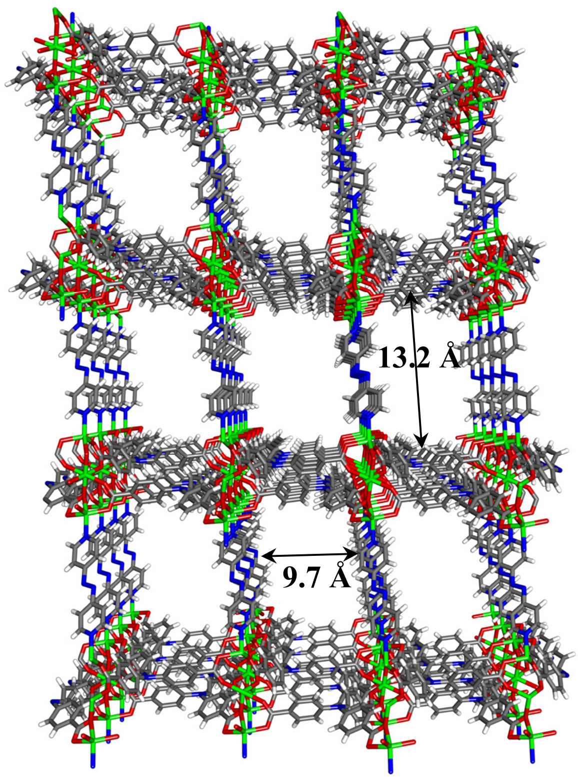

Supplementary Figure 6. One-dimensional rectangle channels along the *b*-axis in an identical net of Zn-**DPA**·$2H_2O$.

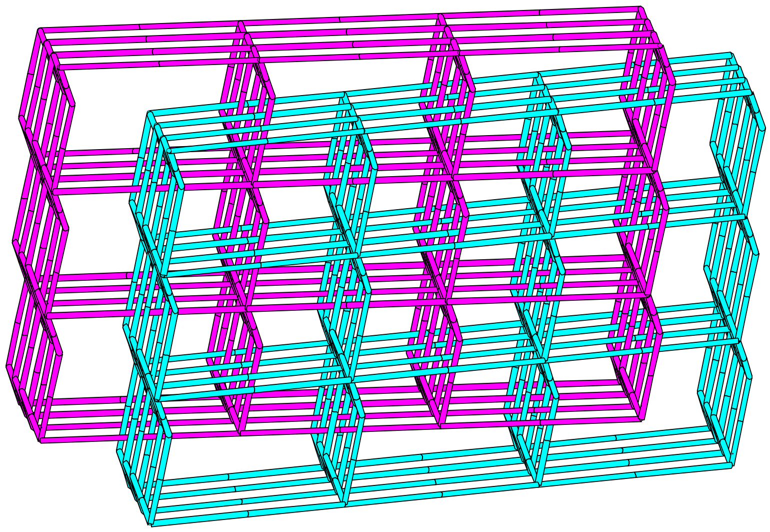

Supplementary Figure 7. View of the 2-fold interpenetrated framework Zn-**DPA**·$2H_2O$ with the $\{(4^3)_2(4^6{\cdot}6^{18}{\cdot}8^4)\}$-**tfz-d** topology.

Supplementary Table 2. Comparison of crystal structures between Zn-**DPA**·$2H_2O$, Zn-**DPA** and Zn-**DPA**·$2CO_2$.

PCP	dihedral angles between the planes (deg)					torsion angles of O_{oxo}-Zn-O-C (deg)	N-C distances in tca^{3-} (Å)
	Zn-O-Zn *vs* phenyl[a]	Zn-O-Zn *vs* pyridine[b]	Zn-O-Zn *vs* O-C-O[c]	O-C-O *vs* phenyl[d]	phenyl *vs* pyridine[e]		
Zn-**DPA**·$2H_2O$	13.35	87.05	11.61	3.23	87.91	-3.08[e] -56.95[f]	1.4140(0.0032)<br>1.4268(0.0034)<br>1.4283(0.0033)
Zn-**DPA**	13.42	88.63	12.18	4.53	89.49	1.30[e] --58.64[f]	1.4153(0.0028)<br>1.4302(0.0029)<br>1.4316(0.0029)
Zn-**DPA**·$2CO_2$	9.26	89.48	12.55	4.49	85.57	-5.26[e] -60.14[f]	1.4123(0.0025)<br>1.4201(0.0027)<br>1.4315(0.0025)

[a] Phenyl rings involving Zn1, Zn2, O2, and O3. [b] Pyridine rings involving Zn1, Zn2, O2, and O3. [c] The plane including Zn1, Zn2, O2, and O3 *vs* the plane including O2, C1 and O3. [d] Phenyl rings involving O2, C1 and O3. [e] O1-Zn1-O3-C1. [f] O1-Zn2-O2-C1.

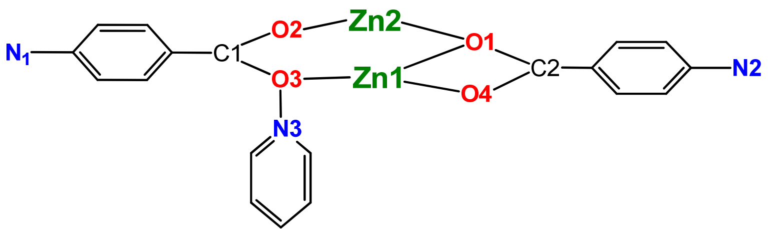

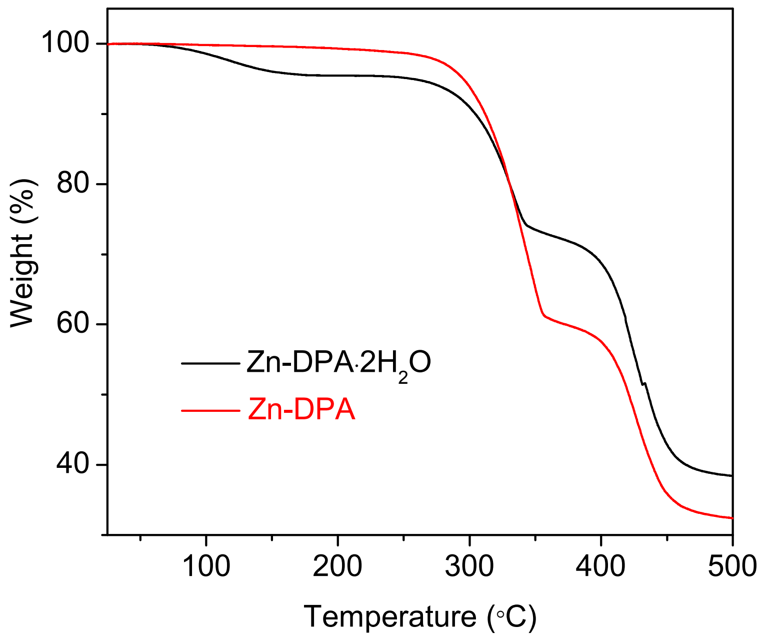

Supplementary Figure 8. Thermogravimetric curves of Zn-**DPA**·2H_2O (red) and Zn-**DPA** (black) under N_2.

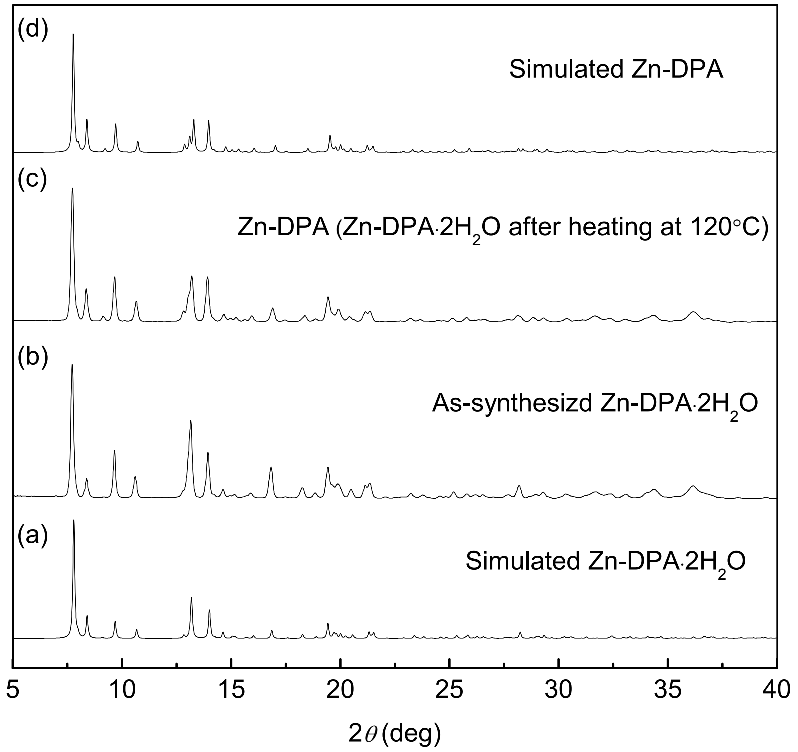

Supplementary Figure 9. PXRD patterns of (a) simulation from single-crystal structure of Zn-**DPA**·2H_2O, (b) the as-synthesized Zn-**DPA**·2H_2O, (c) drying of Zn-**DPA**·2H_2O in *vacuo* at 393 K (Zn-**DPA**) and (d) simulation from single-crystal structure of Zn-**DPA**. Data obtained using Cu *K*α radiation (λ = 1.54 Å).

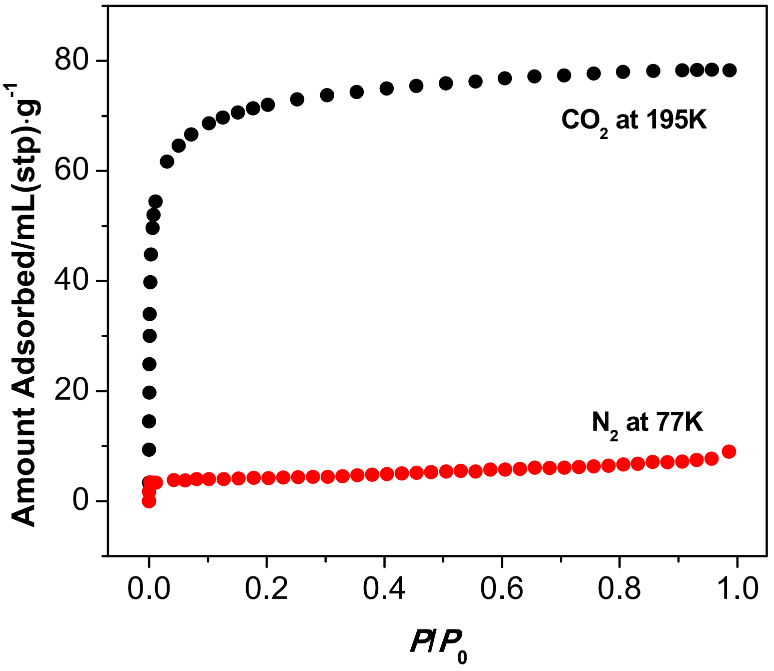

Supplementary Figure 10. Gas sorption isothrems of Zn-**DPA** for CO_2 (195 K) and N_2 (77 K).

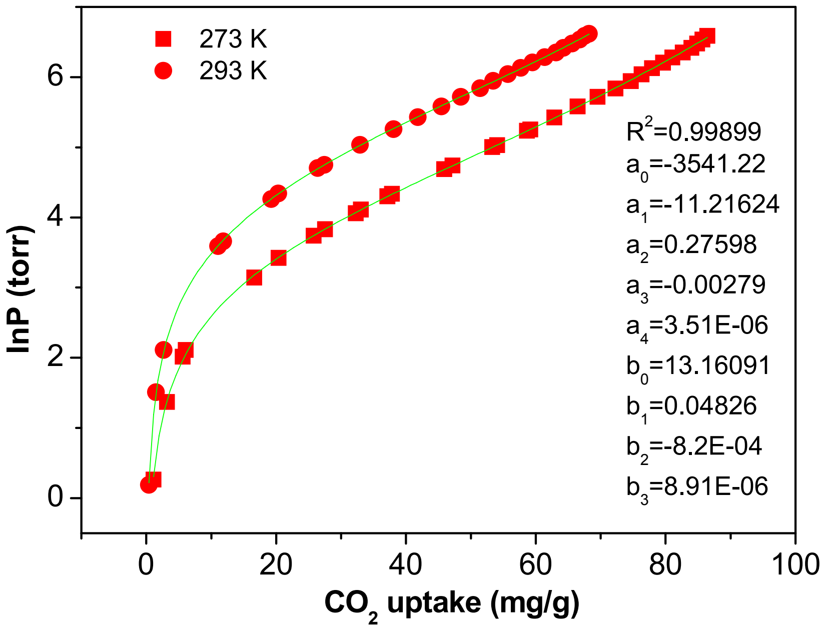

Supplementary Figure 11. The CO_2 isotherms at 273 K and 293 K (symbols) and the virial equation fits (lines) for Zn-**DPA**.

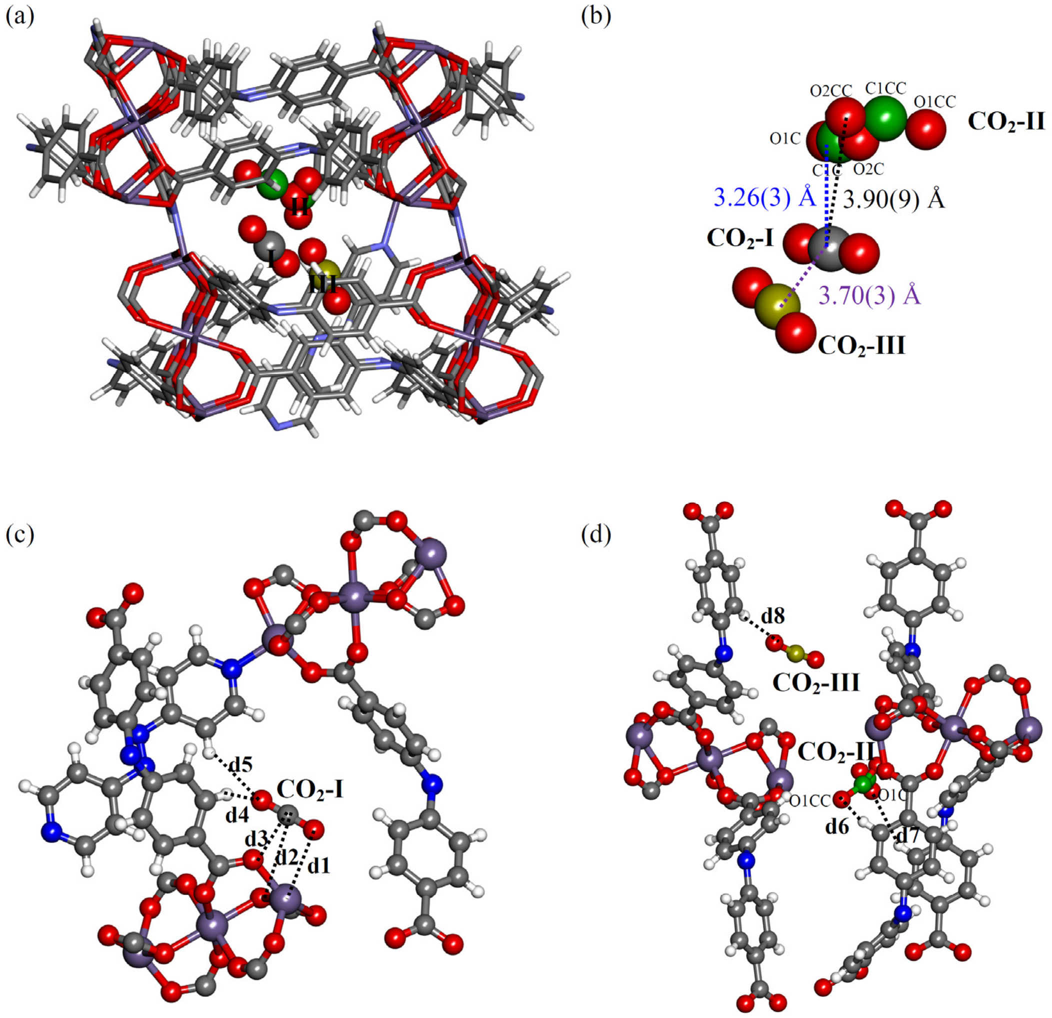

d1 (O···Zn) = 3.17(1) Å

d2 (C···O) = 3.22(9) Å

d3 (C···O) = 3.13(2) Å

d4 (O···H) = 2.88(2) Å

d5 (O···H) = 2.64(7) Å

d6 (O···H) = 2.68(6) Å

d7 (O···H) = 2.84(6) Å

d8 (O···H) = 2.82(1) Å

Supplementary Figure 12. Crystal structure of CO_2-adsorbed Zn-**DPA**·2CO_2. (a) Three crystallographically independent CO_2 molecules are shown trapped in a pore. (b) The cooperative interaction among CO_2 molecules in slipped parallel and T-shaped geometries. (c) The cooperative interaction between CO_2-I and the framework. (d) The cooperative interaction between CO_2-II, CO_2-III and the framework. Atoms except CO_2 molecules are colored as follows: Zn, purple; C, grey; N, blue; O, red.

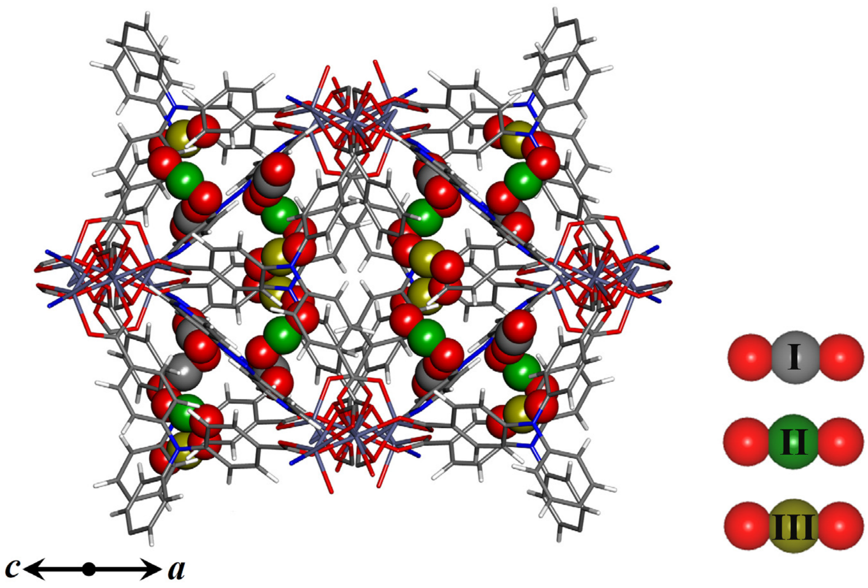

Supplementary Figure 13. CO_2 adsorption positions in Zn-**DPA**. Site I is observed in experiment, while sites II and III are determined through Monte-Carlo simulation followed by geometry optimization using the PBE-D3 method. These adsorption positions are consistent with experimental results obtained from single-crystal X-ray diffraction.

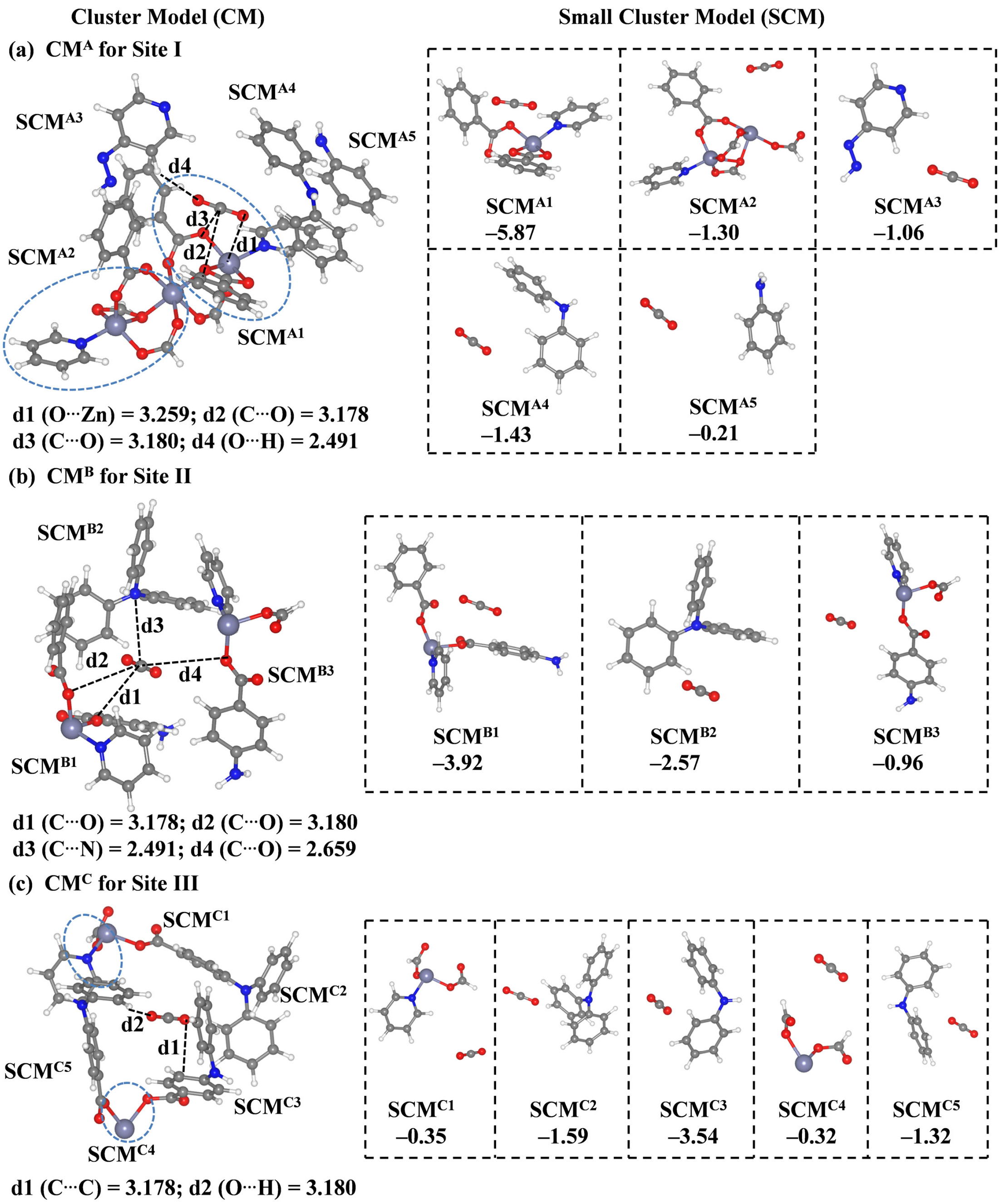

Supplementary Figure 14. Cluster models (CM) and small cluster models (SCM) for sites I (a), II (b) and III (c), where the important geometrical parameters for adsorption of one CO_2 molecule at these sites are presented. The PBE-D3-calculated interaction energies of one CO_2 molecule with each small cluster models are shown below the corresponding structures. Distances and energy values are given in Å and kcal mol^{-1}, respectively.

Supplementary Table 3. PBE-D3-calculated Binding Energy (*BE*, kcal mol^{-1}) of one CO_2 with Zn-**DPA** at sites I, II, and III.

Adsorption Site	I	II	III
BE	–9.28	–7.30	–7.43
E_{INT}(H-G)	–9.88	–7.87	–7.58
E_{DEF}(H)	0.60	0.58	0.15

Supplementary Table 4. PBE-D3-calculated Binding Energy (*BE*, kcal mol^{-1}) of CO_2 with Zn-**DPA** at the sites II and III in the presence of 8 CO_2 molecules at the site I.

Adsorption Site	II	III
BE	–8.75	–8.43
E_{INT}(H-G)	–7.88	–6.81
E_{INT}(G-G)	–1.16	–1.93
E_{DEF}	0.29	0.31

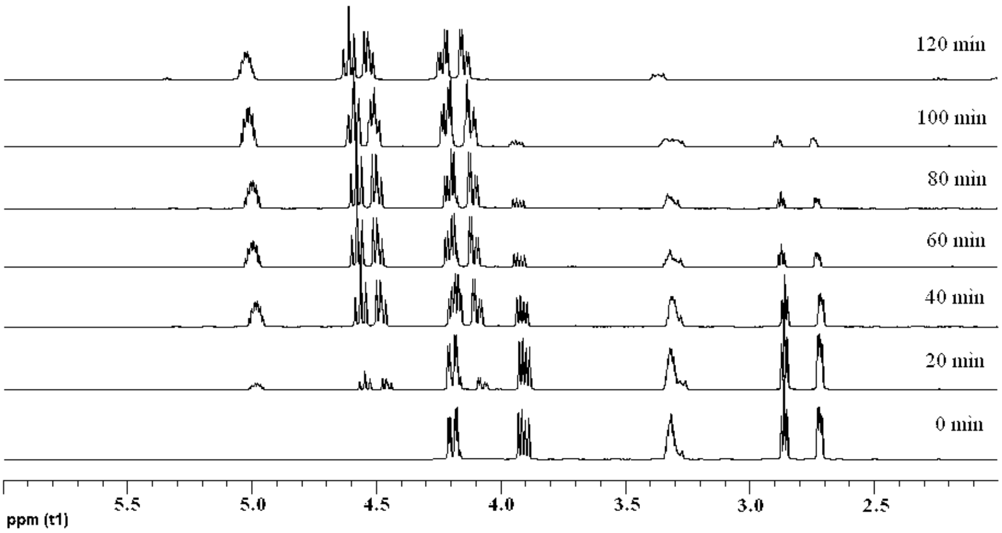

Supplementary Figure 15. ^1H NMR ($CDCl_3$, 400 MHz) monitoring of the cycloaddition reaction progress using catalyst Zn-**DPA**, showing the increasing tendency of the peaks at around 5.02, 4.60, 4.55, 4.22 and 4.16 *ppm*, while the gradual vanish of the peaks at around 4.18, 3.92, 3.32, 2.86 and 2.72 *ppm*, respectively, with the proceeding of the reaction.

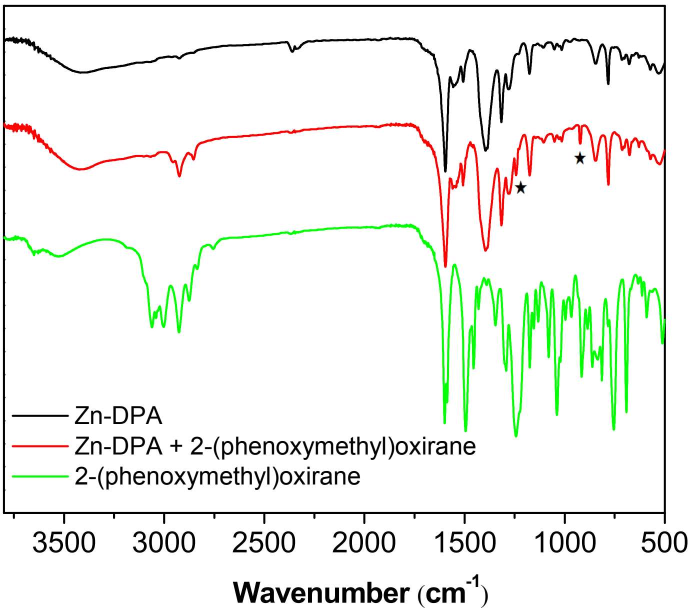

Supplementary Figure 16. IR comparison between Zn-**DPA**, free 2-(phenoxymethyl)oxirane and Zn-**DPA**⊃2-(phenoxy-methyl)oxirane, the emerging peaks at 1275 and 920 cm^{-1} corresponding to the characteristic peaks of $v^{as}_{Ar\text{-}O\text{-}C}$ and $v^{s}_{C\text{-}O\text{-}C}$ in the region marked by black stars.

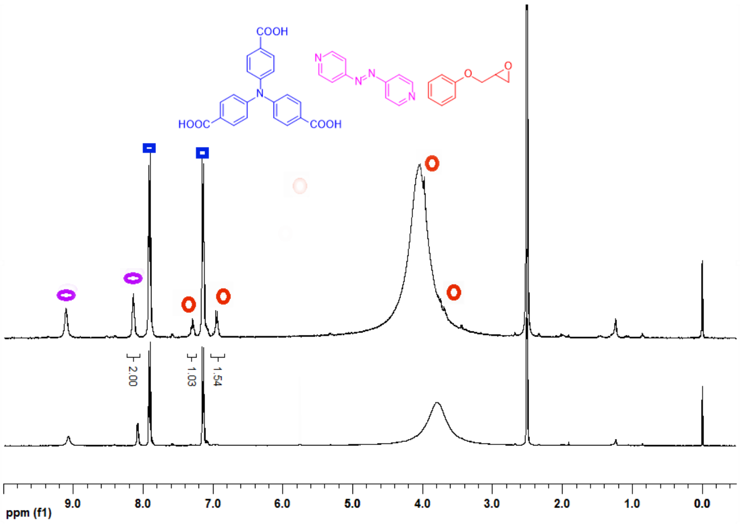

Supplementary Figure 17. ^{1}H NMR spectra of Zn-**DPA** and Zn-**DPA**⊃2-(phenoxymethyl)oxirane dissolved in DMSO-d_6/DCl. Peaks marked with red circles, blue rectangles and purple ovals represent signals of styrene oxide, (E)-1,2-di(pyridin-4-yl)diazene and tca^{3-} ligand, respectively.

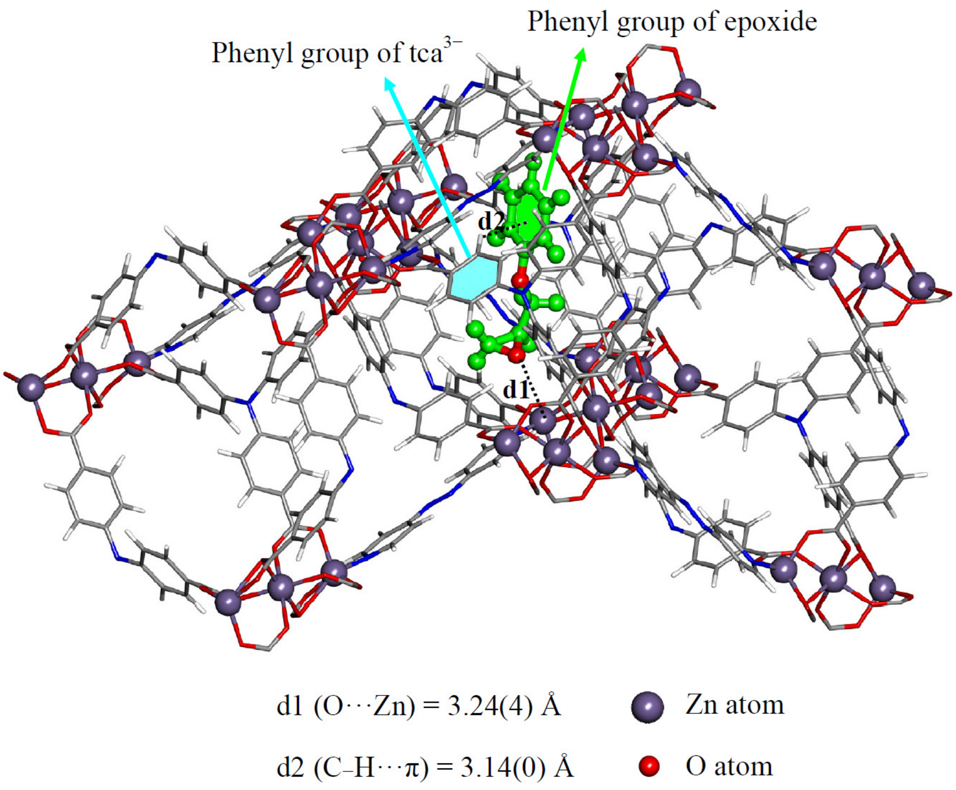

Supplementary Figure 18. Interaction representation between 2-(phenoxymethyl)oxirane and Zn-**DPA** computed by density functional theory calculation.

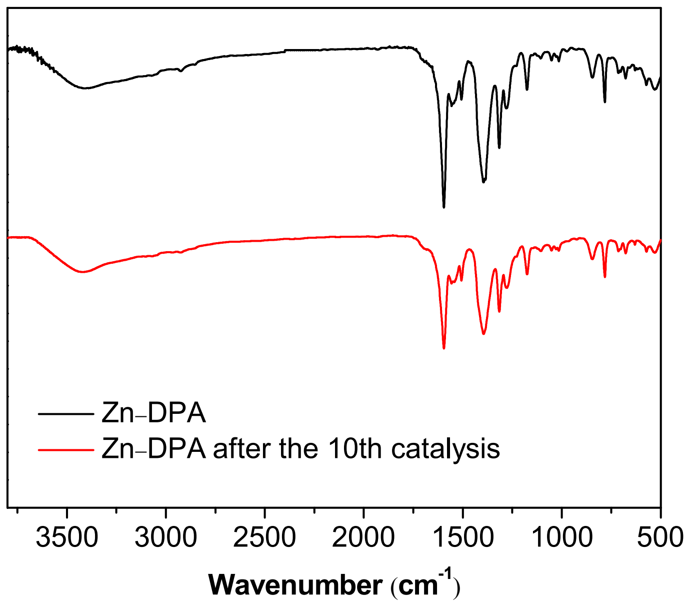

Supplementary Figure 19. IR spectra of Zn-**DPA** and their samples after recycled reactions.

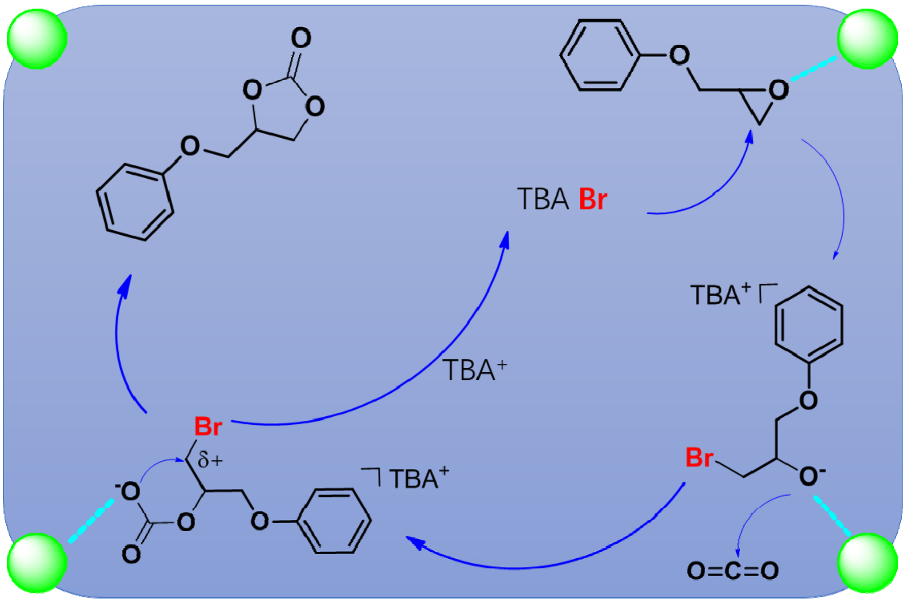

Supplementary Figure 20. The proposed mechanism for the cycloaddition reaction catalyzed by Zn-**DPA** (cyan sphere: open metal site).

Supplementary Table 5. Control experiments of coupling of 2-(phenoxymethyl)oxirane with CO_2.[a]

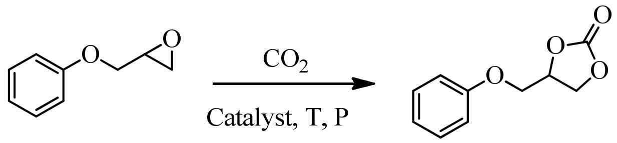

Entry	Catalyst	Co-catalyst	Yield(%)[c]	TON[d]	TOF[e]
1	none	none	*n.d.*	*n.d.*	*n.d.*
2	Zn-**DPA**	none	10	400	200
3	none	TBABr	12		
4[b]	H_3tca	TBABr	12	480	240
5[b]	abp	TBABr	12	960	480
6[b]	$Zn(NO_3)_2 \cdot 6H_2O$	none	11	294	147
7[b]	$Zn(NO_3)_2 \cdot 6H_2O$	TBABr	43	1146	573
8	Zn-**DPA**	TBABr	> 99	4000	2000

[a] Reaction conditions: 2-(phenoxymethyl)oxirane (20 mmol), catalyst (5 μmol, based on $Zn_{1.5}$ cluster) and TBABr (0.3 mmol) under carbon dioxide (1 MPa), 373 K and 2 h.[b] The amount of subcompounts: H_3tca (5 μmol), abp (2.5 μmol), $Zn(NO_3)_2 \cdot 6H_2O$ (7.5 μmol), confirming the same concentration as these units of Zn-DPA. [c] Yield of isolated product was determined from by ^1H NMR spectroscopy. [d] Moles of cyclic carbonate per mole of catalyst. [e] Moles of cyclic carbonate per mole of catalyst per hour.

Supplementary Table 6. MOF-508b[13]-catalyzed coupling of epoxides with CO_2.[a]

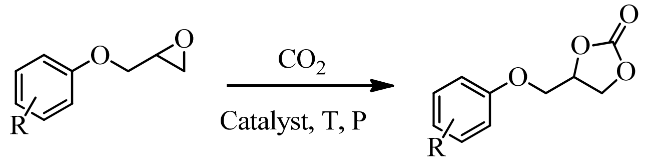

Entry	Epoxides	Yield[b] (%)	TON[c]	TOF[d]
1	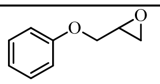	42	2230	1115
3	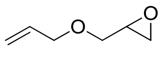	47	2500	1250
4	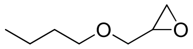	45	2390	1195
5	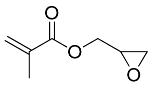	46	2450	1225
6	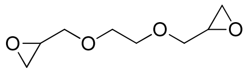	44	2340	1170
7	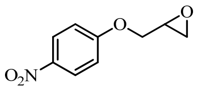	42	2230	1115
8	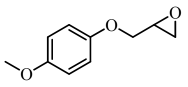	43	2290	1145
9	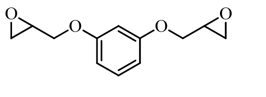	41	2180	1090
10	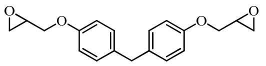	42	2230	1115

[a] Reaction conditions: epoxide (20 mmol), catalyst (3.75 μmol, based on Zn_2 cluster) and TBABr (0.3 mmol) under carbon dioxide (1 MPa), 373 K and 2 h. [b] Yield of isolated product was determined from by ^{1}H NMR spectroscopy. [c] Moles of cyclic carbonate per mole of catalyst MOF-508b. [d] Moles of cyclic carbonate per mole of catalyst MOF-508b per hour.

Supplementary Table 7. Comparison with different PCP catalysts in the cyclic addition of CO_2 and 2-(phenoxymethyl)oxirane.

Entry	Catalyst	P (MPa)	T (℃)	T (hr)	TON	TOF	Ref.
1	1·Mn	2.0	80	4	728	182	[14]
2	BIT−103	3.0	160	24	238	9.9	[15]
3	MOF−5	6.0	4	50	22.4	7.5	[16]
4	Ni−TCPE2	1.0	100	12	1950	162	[17]
5	F-ZIF−90	1.17	120	6	545	90.8	[18]
6	MMPF−18	0.1	r.t.	48	133	2.8	[19]
7	CHB(M)	1.2	120	6	52.8	8.8	[20]
8	ZIF−67	1.0	100	14	37.3	2.7	[21]
9	Ni(salphen)	2.0	80	24	292	12	[22]
10	MMCF−2	0.1	r.t.	48	300.8	6.3	[23]
11	Zn-**DPA**	1.0	100	2	4000	2000	This work
12	Zn-**DPA**	0.1	r.t.	2	320	160	This work

Supplementary Reference:

[1] Frenkel, D. & Smit, B. *Understanding molecular simulations: from algorithms to applications*. Academic Press, San Diego, 2002.

[2] Dubbeldam, D., Calero, S., Ellis, D. E. & Snurr, R. Q. RASPA: molecular simulation software for adsorption and diffusion in flexible nanoporous materials. *Mol. Simul.* **42**, 81–101 (2016).

[3] Rappe, A. K., Casewit, C. J., Colwell, K. S., Goddard III, W. A. & Skiff, W. M. UFF, a full periodic table force field for molecular mechanics and molecular dynamics simulations. *J. Am. Chem. Soc.* **114**, 10024−10035 (1992).

[4] Potoff, J. J. & Siepmann, J. I. Vapor−liquid equilibria of mixtures containing alkanes, carbon dioxide and nitrogen. *AIChE J.* **47**, 1676−1682 (2001).

[5] Manz, T. A. & Limas, N. G. Introducing DDEC6 atomic population analysis: part 1. Charge partitioning theory and methodology. *RSC Adv.*, **6**, 47771−47801 (2016).

[6] Limas, N. G. & Manz, T. A. Introducing DDEC6 atomic population analysis: part 2. Computed results for a wide range of periodic and nonperiodic materials. *RSC Adv.*, **6**, 45727−45747 (2016).

[7] Kresse, G. & Furthmüller, J. Efficiency of ab-initio total energy calculations for metals and semiconductors using a plane-wave basis set. *Comput. Mater. Sci.* **6**, 15−50 (1996).

[8] Kresse, G. & Furthmüller, J. Efficient iterative schemes for *ab initio* total-energy calculations using a plane-wave basis set. *Phys. Rev. B* **54**, 11169−11186 (1996).

[9] Perdew, J. P., Burke, K. & Ernzerhof, M. Generalized gradient approximation made simple. *Phys. Rev. Lett.* **77**, 3865−3868 (1996).

[10] Grimme, S., Antony, J., Ehrlich, J. & Krieg, J. A consistent and accurate *ab initio* parametrization of density functional dispersion correction (DFT-D) for the 94 elements H-Pu. *J. Chem. Phys.* **132**, 154104-1−154104-19 (2010).

[11] Blöchl, P. E. Projector augmented-wave method. *Phys. Rev. B* **50**, 17953−17979 (1994).

[12] Kresse, G. & Joubert, D. From ultrasoft pseudopotentials to the projector augmented-wave method. *Phys. Rev. B* **59**, 1758−1775 (1999).

[13] Chen, B. *et al*. A microporous metal-organic framework for gas-chromatographic separation of alkanes. *Angew. Chem.* **118**, 1418−1421 (2006).

[14] Jiang, W., Yang, J., Liu, Y. Y., Song, S. Y. & Ma, J. F. A Porphyrin-based porous rtl metal-organic framework as an efficient catalyst for cycloaddition of CO_2 to epoxides. *Chem. Eur. J.* **22**, 16991–16997 (2016).

[15] Huang, X. *et al*. Zn-BTC MOFs with active metal sites synthesized *via* a structure-directing approach for highly efficient carbon conversion. *Chem. Commun.* **50**, 2624–2627 (2014).

[16] Song, J., Zhang, Z., Hu, S., Wu, T., Jiang, T. & Han, B. Metal–organic frameworks HKUST-1 as porous matrix for encapsulation of basic ionic liquid catalyst: effect of chemical behaviour of ionic liquid in solvent. *Green Chem.* **11**, 1031–1036 (2009).

[17] Zhou, Z., He, C., Xiu, J. H., Yang, L. & Duan, C. Y. Metal–organic polymers containing discrete single-walled nanotube as a heterogeneous catalyst for the cycloaddition of carbon dioxide to epoxides. *J. Am. Chem. Soc.* **137**, 15066–15069 (2015).

[18] Jose, T., Hwang, Y., Kim, D. W., Kim, M.-Il & Park, D. W. Functionalized zeolitic imidazolate framework F-ZIF-90 as efficient catalyst for the cycloaddition of carbon dioxide to allyl glycidyl ether. *Catal. Today* **245**, 61–67 (2015).

[19] Gao, W. Y., Tsai, C. Y., Wojtas, L., Thiounn, T., Lin, C. C. & Ma, S. Q. Interpenetrating metal–metalloporphyrin framework for selective CO_2 uptake and chemical transformation of CO_2. *Inorg. Chem.* **55**, 7291–7294 (2016).

[20] Kathalikkattil, A. C., Kim, D. W., Tharun, J., Soek, H. G., Roshan, R. & Park, D. W. Aqueous-microwave synthesized carboxyl functional molecular ribbon coordination framework catalyst for the synthesis of cyclic carbonates from epoxides and CO_2. *Green Chem.* **16**, 1607–1616 (2014).

[21] Mousavi, B., Chaemchuen, S., Moosavi, B., Luo, Z., Gholampour, N. & Verpoort, F. Zeolitic imidazole framework-67 as an efficient heterogeneous catalyst for the conversion of CO_2 to cyclic carbonates. *New J. Chem.* **40**, 5170–5176 (2016).

[22] Ren, Y., Shi, Y., Chen, J., Yang, S., Qi, C. & Jiang, H. Ni(salphen)-based metal–organic framework for the synthesis of cyclic carbonates by cycloaddition of CO_2 to epoxides. *RSC Adv.* **3**, 2167–2170 (2013).

[23] Gao, W. Y. *et al*. Crystal engineering of an nbo topology metal-organic framework for chemical fixation of CO_2 under ambient conditions. *Angew. Chem. Int. Ed.* **53**, 2615–2619 (2014).
